# Financial burden and health-seeking behaviors related to chronic diseases under the National Health Insurance Scheme in Bolikhamxay Province, Lao PDR: a cross-sectional study

**DOI:** 10.1186/s12939-022-01788-0

**Published:** 2022-12-16

**Authors:** Tomoo Ito, Sengchanh Kounnavong, Chiaki Miyoshi

**Affiliations:** 1 Bureau of International Health Cooperation, National Center for Global Health and Medicine, 1-21-1 Toyama Shinjuku-ku, Tokyo, 162-8655 Japan; 2 Lao Tropical and Public Health Institute, Ministry of Health, Samsenethai Road, Ban Kaognot, Sisattanack District, Vientiane Capital, Lao PDR

**Keywords:** Chronic disease, Finances, Health behavior, Health insurance, Universal health coverage

## Abstract

**Background:**

Chronic diseases pose a serious threat to health and longevity worldwide. As chronic diseases require long periods of treatment and may become serious conditions, the ensuing financial burden is often worse than that for non-chronic diseases. In 2016, the Lao PDR implemented the National Health Insurance (NHI) system, which covers select provinces. However, data on health service accessibility and the financial burden on households, especially those with chronically ill members covered by the NHI, are scarce.

**Methods:**

This study used a cross-sectional design. Data collection was conducted in Bolikhamxay province (population = 273,691), from January 15 to February 13, 2019. In total, 487 households, selected through stratified random sampling, were surveyed via questionnaire-based interviews. Healthcare service usage and financial burden were examined.

**Results:**

A total of 370 households had at least one member with self-reported health issues within the last 3 months prior to the interview, while 170 had at least one member with a chronic condition. More than 75% of the households accessed a health facility when a member experienced health problems. The majority of households (43.2%) spent the maximum value covered by the NHI, but households in the second largest group (21.4%) spent 10 times the maximum value covered by the NHI. The prevalence of catastrophic health expenditure (i.e., health-related expenditure equivalent to > 20% of total income) was 25.9% (20% threshold) and 16.2% (40% threshold). Through logistic regression, we found that the major factors determining financial catastrophes owing to health problems were household members with chronic illness, hospitalization, household poverty status, household size (for both the 20 and 40% thresholds), visiting a private facility (20% threshold), and distance from the province to the referral hospital (40% threshold).

**Conclusions:**

The NHI system has had a positive effect on households’ access to health facilities. However, catastrophic health expenditure remains high, especially among chronically ill patients. Facilities under the NHI system should be improved to provide more services, including care for chronic conditions.

## Background

Chronic diseases (e.g., major non-communicable diseases [NCDs] such as cardiovascular diseases, cancer, chronic respiratory diseases, and diabetes) often lead to disabilities, while also posing a serious threat to health and longevity. Chronically ill patients have a higher risk of developing comorbid diseases, which worsen their health condition over time [1]. The burden of such diseases is more serious in low- and middle-income countries because of the myriad challenges in appropriate treatment, prevention, and early detection, compared with high-income countries [2, 3]. Several studies conducted in the Lao PDR have reported a high prevalence of morbidity and chronic diseases [4–7]. As chronic diseases must be treated over long periods, the ensuing financial burden is often worse than that for non-chronic diseases, which are commonly treated over short periods. Furthermore, in the advanced stages of chronic disease, more intensive and advanced treatments are needed to ensure patients’ survival. In such cases, higher treatment costs are common, which reduces health services accessibility for patients in the advanced stages of a disease. Further, even when patients finally receive treatment, they incur catastrophic health expenditure (CHE). Moreover, in most countries, the poorest people have the highest risk of developing chronic diseases and are the least able to cope with the financial consequences [8]. Thus, providing people with timely access to affordable healthcare is crucial. Nevertheless, reports show that 1.3 billion people worldwide are unable to access affordable and effective healthcare services [9].

Universal health coverage (UHC) is defined as a system in which all individuals and communities have access to health services without suffering financial hardship; this includes the full spectrum of essential and quality health services, ranging from health promotion to prevention, treatment, rehabilitation, and palliative care [10]. Many countries are working to implement UHC systems. In this context, financial protection plays a critical role [10–13]. Some studies posit that one of the barriers to healthcare access comprises financial burden—out-of-pocket (OOP) payments for treatment [14–16].

The government of the Lao PDR has attempted to address these issues and facilitate the implementation of UHC through the introduction of the National Health Insurance (NHI) system, which was launched in select provinces in 2016 [17, 18] and may soon be expanded to all provinces. Under the NHI, the payment system for health services has been simplified; healthcare visits require only a small, fixed co-payment at the facility level, regardless of the administered treatment (Table 1).

**Table 1:** Overview of the National Health Insurance system in the Lao PDR [18]

Population coverage	All people in the target areas covered by the NHI who are not yet registered in the system. Eligible members include individuals in the selected provinces’ family books or with a corresponding identification, as well as individuals with a certificate from the village chiefs of the target provinces.
Available facilities	NHI members can use contracted facilities directly. However, when they use other facilities, they require a referral letter from the contracted health facility.
Co-payment	Under the NHI, the payment system for health services has been simplified: healthcare visits require only a small, fixed co-payment at the facility level, regardless of the administered treatment. For instance, outpatients at provincial hospitals (i.e., referral hospitals), district hospitals, and health centers must submit flat co-payments of 15,000 LAK (0.93 USD), 10,000 LAK (0.62 USD), and 5000 LAK (0.31 USD), respectively. For inpatients, the co-payments at both provincial and district hospitals amount to 30,000 LAK (1.87 USD) and 5000 LAK (0.31 USD), respectively, at health centers. The NHI package officially includes a range of services, such as acute and long-term care (including palliative care), and there are no cost limitations.
Service coverage	NHI members (insurers) can use all healthcare services without specific fees at public healthcare facilities, including drug fee, treatment service fee, medical equipment fee, diagnostic fee, document fee, patient room fee, and other medical and non-medical fees.
Funding resources	Government budget, donor support, private organizations or individuals, and NHI-generated earnings.

*NHI* National Health Insurance

In implementing the NHI, the Lao PDR government aimed to ensure smooth access to healthcare and prevent CHE. Some studies have investigated the NHI’s impact in the Lao PDR [19, 20]; however, data on health service accessibility and CHE for households covered by the NHI are scarce, thereby highlighting another research gap—this is especially true of households with chronically ill patients. To bridge these gaps, in this study, we aimed to clarify citizens’ health-seeking behaviors, the prevalence of CHE, and the factors influencing CHE. To this end, we analyze households in Bolikhamxay province, including those with chronically ill patients. We chose this province because the NHI has been in operation here for more than 2 years. To help support the nationwide realization of NHI goals, we aimed to provide data to clarify potential pathways to more well-informed decision-making among managers and policymakers regarding the improvement of health insurance coverage and affordability.

## Methods

### Study design, area, and data collection method

Using a cross-sectional design, we investigated the health-seeking behaviors, prevalence of health problems, and related financial burden in households in Bolikhamxay province over the past 3 months. According to the latest data from this province, its total population is 273,691; it covers an area of 14,863 km^2^ (i.e., 5739 mi^2^), with a population density of 18/km^2^ [21]. It shares borders with Xiengkhouang Province to the northwest, Vietnam to the east, Khammouane Province to the south, and Thailand to the west. Bolikhamxay province consists of 7 districts (Pakxanh, Thaphabath, Pakkading, Borikhan, Viengthong, Xaychamphone, and Khamkeuth); it has 1 provincial hospital, 6 district hospitals, and 40 health centers [22].

Researchers working in the Lao Tropical and Public Health Institute were trained in interviewing procedures and conducted questionnaire-based interviews with the sampled households from January 15 to February 13, 2019. All interviews were conducted with the head of each household, who provided information about their family members, occasionally consulting with other family members about the same.

### Sample size and household selection process

The sampling unit was a household, defined as a group of individuals living together, usually comprising parents and children, and sometimes including grandparents and uncles. To calculate the acceptable sample size for this study, we referred to a previous study on CHE conducted in Vietnam, a neighboring country with a similar political system, which showed a 17.4% CHE prevalence (20% threshold) in 2004 [23]. We also considered a margin of error of ±5, significance at the 99% confidence level, and Z = 2.57. The total number of households in Bolikhamxay province was 54,738—a number obtained by dividing the total population (273,691) [21] by the average number of members in a household (5) [21]. This yielded a sample size of 384 households. To address the possibility of withdrawal, approximately 25% more households were added to the sample, resulting in a final sample size of 480 households.

$$\textbf{Sample}\ \textbf{size},\textbf{n}=\textbf{N}\ast \frac{\frac{{\boldsymbol{Z}}^{\textbf{2}}\ast \boldsymbol{p}\ast \left(\textbf{1}-\boldsymbol{p}\right)}{{\boldsymbol{e}}^{\textbf{2}}}}{\left[\boldsymbol{N}-\textbf{1}+\frac{{\boldsymbol{Z}}^{\textbf{2}}\ast \boldsymbol{p}\ast \left(\textbf{1}-\boldsymbol{p}\right)}{{\boldsymbol{e}}^{\textbf{2}}}\right]}$$

where the margin of error is ±5, significant at the 95% confidence level, Z = 1.96, the number of households in the province is 54,738, and the estimated proportion of households incurring CHE is 17.4%.

Using a stratified systematic method, the households were sampled as follows. First, we selected three districts, including the provincial capital district (Pakxan). The other two were chosen based on their distance (in km) from Pakxan; the first, Thaphabad, was 50 km away, while the second, Pakkading, was 70–100 km away. As there is only one medical facility at the provincial level in Pakxan, we classified districts into three categories (near, halfway, and far from the capital), assuming that distance from the capital would likely have a strong impact on healthcare access for patients who needed advanced medical intervention. Second, based on the National Statistical Office’s definition of rural areas, we divided the villages in each district into three groups (urban village, rural village with road, and rural village without road). From each group of villages, we randomly selected five villages in each district: one urban village, two rural villages with roads, and two rural villages without roads. We adopted this approach because we assumed that urbanization and the availability of roads would greatly affect general healthcare access within each district. Third, we randomly selected 32–33 households from each village, using the village registration book, with equal intervals on the lists. To ensure the minimum sample size acceptable for this study, upon not acquiring sufficient valid responses from the first group of selected households, we analyzed a novel batch of interviews with the next households on the list, and this procedure was repeated until we reached an acceptable number of valid responses from 32 households per village.

### Definitions

#### Chronic disease

Prior research has posited that differences in the conceptualization of the term “chronic disease” occur largely due to the research data and the research field of lead authors [24]. As we wanted to focus our investigation on all diseases that involved relatively longer treatment periods (to analyze their possible financial burden), regardless of disease course, we chose to simplify the definition of chronic disease. In this study, a chronic disease refers to a disease that a patient has been diagnosed with for more than 3 months at the time of the survey [25]. In this study, we classified diseases into chronic and non-chronic, according to the duration of the condition. For example, if patients had an NCD but they were cured and stopped receiving treatment within the last 3 months, said disease was not categorized as chronic.

#### Catastrophic health expenditure (CHE)

This is defined as an event in which a household’s medical expenditure exceeds a certain threshold according to the household’s capacity to pay. In this study, to calculate CHE, we adopted the proportionality of income approach [26], where we considered the total monthly OOP spending as a proportion of monthly income, or the proportion of household OOP spending for healthcare greater than the CHE configured in pre-specified proportions. For this study, the data on total household income and total health expenditure per month were obtained from the questionnaire. The total health expenditure per month was calculated via the following procedure. First, in the questionnaire, we asked each household head about the health services their household used and the associated costs in the 3 months preceding the interview. Further, we explained that total health expenditure was defined as the average health expenditure per month, which comprises the sum of all expenses related to medicines, transportation to and from health facilities, consultation and treatment costs, laboratory tests and diagnostic fees, hospitalization fees, cost of visits to traditional healers, and other health-related expenditures during the last 3 months, which was then divided by three. Subsequently, CHE was calculated as the average health expenditure per month in the household divided by household’s total monthly income; the latter value was also obtained during the interview.

Our literature review showed a lack of consensus regarding the thresholds indicating CHE; specifically, we observed thresholds varying from 5 to 40% of total household income [26–31]. While there is no consensus regarding the CHE threshold, in this study, we employ the threshold proposed by Xu et al., who define health expenditure as catastrophic if a household’s financial contributions to health equal and/or exceed 40% of non-food expenditure or capacity to spend [29]. However, Rashidian et al. contend that the appropriate cut-off points for the proportion of OOP health expenses to total expenditure and the proportion of health expenditure to ability to pay is 20% [30]. Considering these two previous studies, we set a healthcare expenditure of 20% of the total household income as the threshold for CHE and an expenditure of 40% as the threshold for serious CHE.

### Questionnaire structure and independent variables

The questionnaire was developed by drawing on a number of studies on CHE, healthcare utilization, and chronic disease, as well as previous household surveys [26, 31–37]; the relevant items were adapted to the Lao PDR’s context. We divided the questions into seven sections: a) household composition and demographic characteristics (total monthly income, educational level of the household head, and distance between the household and the nearest medical facility); b) self-reported health problems of household members in the last 3 months (including a variety of health issues ranging from mild to severe, including injuries); c) health-seeking behavior (comprising self-medication and healthcare services—including medical consultations—availed by household members during the preceding 3 months); and e) health services availed and associated costs in the 3 months preceding the interview. Total health expenditure was determined as the sum of all spending on medicines, transportation to and from health facilities, consultation and treatment costs, laboratory tests and diagnostic fees, hospitalization fees, cost of visits to traditional healers, and other health-related expenditures during the last 3 months—converted to 4 weeks, following a previous study [26]. To finalize the questionnaire, a pilot study was carried out with 100 households in Khammouane province (which borders the south of Bolikhamxay Province) to evaluate the accuracy, rigor, and communicability of the questionnaire.

Following previous studies, households were categorized into four groups, in descending order of income, as richest, rich, poor, and poorest. The education level of the head of the household was categorized as under or above primary education. Household size was defined as the number of individuals in a household (< 5 people or ≥ 5 people). Place of residence was defined as the distance from the nearest health facility treating patients with non-severe illnesses (< 5 km or ≥ 5 km) and the provincial-level referral facility treating patients with more severe illnesses (< 10 km or ≥ 10 km). The type of facility visited was defined as public or private. The type of illness suffered was defined as chronic or non-chronic.

### Data analysis

SPSS™ version 25 (IBM Corp., Armonk, NY, USA) software was used for statistical analysis. A descriptive analysis was undertaken to understand household background, occurrence and type of health problems, health-seeking behavior (for households and individuals, separately), and household OOP payments. Chi-square statistics were used to compare non-chronically and chronically ill patients’ demographic variables and the different types of facilities used when incurring CHE. Mann-Whitney t-tests were used to compare OOP expenses between the different types of facilities. A logistic regression (logit) model was used to predict the probability of CHE. Based on the literature, we first assumed that the type of illness and treatment episodes had an impact on households with CHE. We expected that chronic illness, hospitalization, treatment in a private facility, and treatment in a provincial-level referral hospital would be associated with high healthcare expenditure.

The second group of variables comprised household characteristics, which included household size, educational level of the head of household, and distance from the nearest health facility as well as the nearest provincial-level referral hospital. We also included households’ economic status [37–39]. All these variables were entered into the logit model, using the forward stepwise entry function in SPSS; if the probability of a variable’s score statistic was < 0.05, it was included; conversely, it was removed if the probability was > 0.1. The stepwise entry-removal of the various explanatory variables allowed the identification of those that had a statistically significant influence on the probability of determining CHE. The following variables were included in the model as categorical variables: a) households that incurred expenditure for treating chronic illness and hospitalization (20 and 40% threshold), b) households in which at least one member visited a private facility (20% threshold), c) income groups (20 and 40% thresholds), d) household distance from the nearest provincial-level referral hospital (40% threshold), and e) household size (20 and 40% threshold). The probability of CHE was calculated using the logit model [40], and the model’s goodness-of-fit was assessed via the Hosmer-Lemeshow test [41].

## Results

### Household demographics and disease prevalence in households

**Fig. 1:**
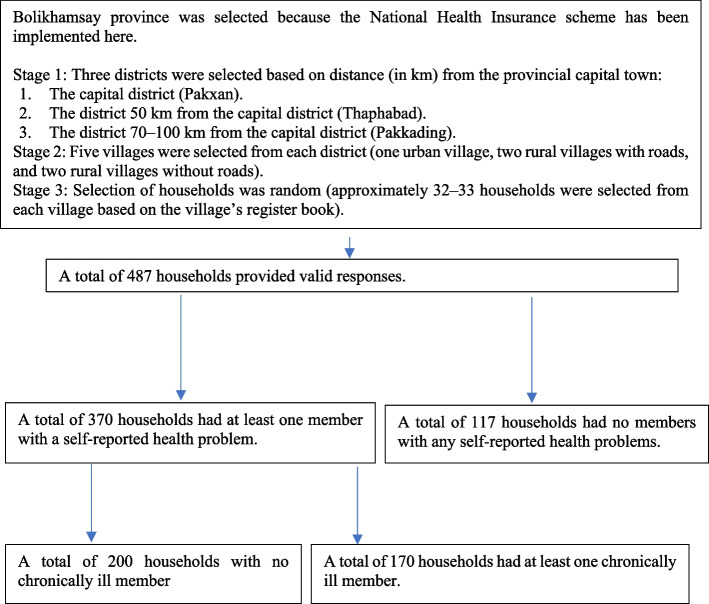
Flow of sampling procedure

All 487 households in Bolikhamxay province agreed to participate in the interviews (Fig. 1).

The results showed that 28.7% of households had an income < 1,000,000 LAK (62.2 USD)/month. Conversely, the richest households, with an income > 3,500,000 LAK (217.7 USD)/month, accounted for 22.4% of all households. Further, 56.9% of the households had ≤ four members, while 49.7% had a primary education level. Moreover, 73.9% had a distance < 5 km from the nearest medical facility, while 80.5% of the households had a distance ≥10 km from the top referral hospital in the province (Table 2).

**Table 2:** Household demographics

	N (% in total households)	N (% of households with at least one family with health problem)
	*N* = 487	*N* = 370
Total household income per month		
Richest (25%). Above 3,500,000 LAK (217.7 USD)/ month	109 (22.4%)	95 (25,7%)
Rich (25%). 2,000,000 LAK (124.4 USD)–3,500,000 LAK (217.7 USD)/month	102 (20.9%)1	70 (18.9%)
Poor (25%). 1,000,000 LAK (62.2 USD)–2,000,000 LAK (124.4)/ month	112 (23.0%)	86 (23.2%)
Poorest (25%). Under 1,000,000 LAK (62.2 USD)/month	140 (28.7%)	98 (26.5%)
Total number of household members		
1–4	277 (56.9%)	192 (51.9%)
> 5	210 (43.1%)	178 (48.1%)
Education of the household head		
Primary education	242 (49.7%)	186 (50.3%)
Above primary education	200 (41.1%)	149 (40.3%)
Distance from the nearest health facility		
< 5 km	360 (73.9%)	276 (74.6%)
≥ 5 km	127 (26.1%)	94 (25.4%)
Distance from the provincial hospital (top referral hospital)		
< 10 km	95 (19.5%)	78 (21.1%)
≥ 10 km	392 (80.5%)	292 (78.9%)

### Disease prevalence and health-seeking behaviors in households

Of the 487 households, 370 (76.0%) had at least one member with a health problem in the past 3 months. Among these, 170 (45.9%) had at least one member with a chronic condition, 79.2% used some kind of medical facility in the past 3 months, 55.4% used a public facility, 20.3% used a private facility, and 8.6% visited a foreign medical facility. Further, 31.6% had experienced hospitalization and 13.8% visited a provincial top referral hospital (Table 3).

**Table 3:** Households’ disease prevalence and health-seeking behaviors

	N (% of total households)	N (% of households with at least one family with health problems)
	*N* = 487	*N* = 370
Households’ health problems		
Households with at least one member with any self-reported health problem	370 (76.0%)	370 (100%)
Households with at least one non-chronically ill member	241 (49.5%)	241 (65.1%)
Households with at least one chronically ill member	170 (34.9%)	170 (45.9%)
Households’ health seeking behaviors		
Households with at least one member who visited a health facility	293 (60.2%)	293 (79.2%)
Households with at least one member who visited a public facility	205 (42.1%)	205 (55.4%)
Households with at least one member who visited a provincial top referral hospital	51 (10.5%)	51 (13.8%)
Households with at least one member who visited a private facility (including foreign facilities) for treatment	75 (15.4%)	75 (20.3%)
Households with at least one member who visited a foreign facility for treatment	32 (6.6%)	32 (8.6%)
Households with at least one member who was hospitalized for more than one night	117 (24.0%)	117 (31.6%)

### Individual disease prevalence and health-seeking behaviors among non-chronically ill and chronically ill patients

The 487 surveyed households had 2692 members. Among these, 375 (13.9%) members reportedly experienced non-chronic illnesses in the past 3 months, while 198 (7.4%) were chronically ill. Approximately 41.9% of non-chronic and 40.9% of chronically ill patients were male. Further, chronically ill patients were older than non-chronically ill patients, and the difference was statistically significant (medium [25–75%]; 56 [46–62], 33 [8–54]; *p* <  0.0001) (Table 4).

**Table 4:** Comparison between non-chronically and chronically ill patients’ demographic characteristics

	Non-chronically ill patients	Chronically ill patients	*p*-values
	*N* = 375	*N* = 198	
Age (years)	33 (8–54)	56 (46–62)	< 0.0001
Gender			0.82
Male	157 (41.9%)	81 (40.9%)	
Female	218 (58.1%)	117 (59.1%)	
What did they do to address their condition?			
Did nothing	30 (8.0%)	0 (0.0%)	< 0.0001
Took care of themselves	83 (22.1%)	42 (21.2%)	0.80
Visited a health center	150 (40.0%)	21 (10.6%)	< 0.0001
Visited a district hospital	41 (10.9%)	37 (18.7%)	0,01
Visited a provincial top referral hospital	29 (7.7%)	69 (34.8%)	< 0.0001
Visited a private facility	54 (14.4%)	37 (18.7%)	0.19

The percentage of non-chronically ill patients (40.0%) who visited health centers for treatment was higher than that of chronically ill patients (10.6%), and the difference was statistically significant (*p* <  0.001). The percentage of chronically ill patients (34.8%) who visited the provincial top referral hospital for treatment was higher than that of non-chronically ill patients (7.7%), and the difference was statistically significant (*p* <  0.0001). The percentage of chronically ill patients (18.7%) who visited private facilities for treatment was higher than that of non-chronically ill patients (14.4%), but the difference was not statistically significant (*p* = 0.19) (Table 4).

### Health facility access by income group

Even in the lowest-income group, more than 85% of the households visited healthcare facilities (Table 5).

**Table 5:** Households that visited healthcare facilities or self-medicated when a family member had a health problem, by household income

	Richest: Above 3,500,000 LAK (217.7 USD)/ month *N* = 95	Rich: 2,000,000 LAK (124.4 USD)–3,500,000 LAK (217.7 USD)/month *N* = 69	Poor: 1,000,000 LAK (62.2 USD)–2,000,000 LAK (124.4 USD)/month *N* = 85	Poorest: Under 1,000,000 LAK (62.2 USD)/month *N* = 98
Households that at least one member visited healthcare facilities	78 (82.1%)	52 (75.4%)	78 (91.8%)	85 (86.7%)
Households that at least one member self-medicated	32 (33.7%)	20 (29.04%).	10 (11.8%)	13 (13.3%)

### Distribution of total monthly household health expenditure and monthly health cost/monthly income (%)

**Fig. 2:**
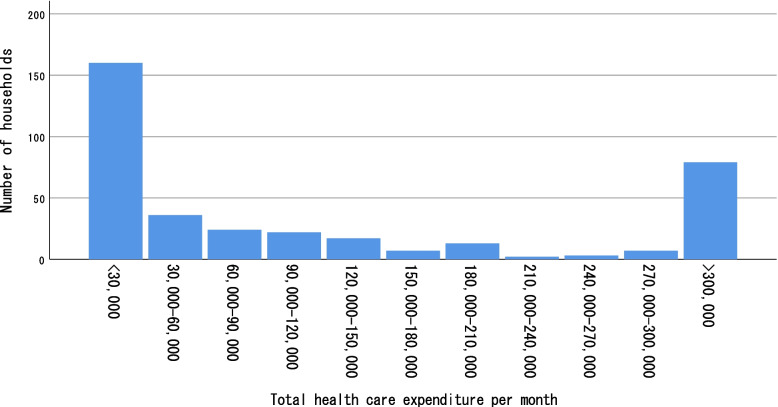
Households’ monthly out-of-pocket healthcare expenditure, divided into 30,000 LAK brackets until reaching > 300,000 LAK

Households with at least one member with any self-reported health problem had a medium OOP health expenditure of 100,000 LAK (6.22 USD; interquartile range, 25,000 LAK [1.6 USD] to 423,750 LAK [26.4 USD]) per month. The majority of households (43.2%) spent less than 30,000 LAK (1.9 USD) per month on healthcare, which is the maximum value covered by the NHI. The distribution of the other expenditure values was polarized; in the second largest group, households spent more than 300,000 LAK (18.7 USD) per month on healthcare, which is 10 times the maximum value covered by the NHI (21.4%; Fig. 2). The OOP health expenditure of households that used only public facilities for treatment (60,000 LAK [3.8 USD]; interquartile range: 20,000 LAK [1.2 USD] to 250,000 LAK [15.6 USD]) and other alternatives (e.g., private facilities or self-medication; 160,000 LAK [10.0 USD]; interquartile range: 50,000 LAK [3.1 USD] to 762,500 LAK [47.42 USD]) was statistically different (*p* <  0.0001; Table 6).

**Table 6:** Out-of-pocket payment and catastrophic health expenditure prevalence comparison between households that visited public facilities and those that used other alternatives for treatment

	Total *N* = 370	Households that only visited public facilities for treatment *N* = 189	Households that used private facilities or performed self-medication *N* = 181	*p*-value
Out-of-pocket health expenditure per month (interquartile range; as LAK)	100,000 (interquartile range: 25,000–423,750)	60,000 (interquartile range: 20,000–250,000)	160,000 (interquartile range: 50,000–762,500)	< 0.0001
Out-of-pocket health expenditure/income (20% threshold)	96 (25.9%)	39 (20.6%)	57 (31.5%)	< 0.0001
Out-of-pocket health expenditure/income (40% threshold)	60 (16.2%)	21 (11.1%)	39 (21.5%)	< 0.0001

**Fig. 3:**
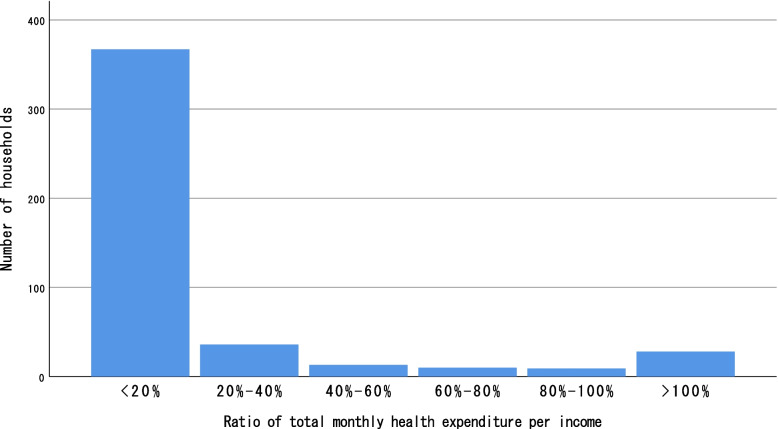
Households’ out-of-pocket health expenditure-to-income ratio, divided into 20% brackets until reaching > 100%

In total, 96 (25.9%) households met the 20% threshold for CHE, while 60 (16.2%) met the 40% threshold. Households that incurred CHE and used only public facilities for treatment differed statistically (*p* <  0.0001) from households that used private facilities (Table 6, Fig. 3).

### Factors influencing catastrophic health expenditure

The logistic regression analysis revealed a wide range of determinants associated with an increased risk of CHE (Tables 6 and 7). It revealed that the odds of incurring CHE were 3.720 (95% CI, 2.051–6.704, *p* <  0.0001; 20% threshold) and 4.354 (95% CI, 2.138–8.866, *p* <  0.0001; 40% threshold) for households with chronically ill members and 2.355 (95% CI, 1.390–4.620, *p* <  0.0001; 20% threshold) and 3.367 (95% CI, 1.702–6.663, *p* < 0.0001; 40% threshold) times higher for households with hospitalized members, respectively. The poorest households were 4.002 (95% CI, 1.821–-8.792, *p* = 0.0001; 20% threshold) and 3.361 times (95% CI, 1.273–8.872, *p* = 0.014; 40% threshold) more likely to incur CHE when compared with the richest ones. Thus, as household monthly income decreased, the probability of incurring CHE increased. Finally, the odds of incurring CHE were 2.466 (95% CI, 1.310–4.640, *p* = 0.005) times higher for households that underwent treatment in private facilities (20% threshold) and 4.587 (95% CI, 1.690–13.558) times higher for households more than 10 km away from the provincial hospital (40% threshold). Households with fewer than five members were 2.127 (95% CI, 1.182–3.829, *p* = 0.012; 20% threshold) and 2.669 (95% CI, 1.321–5.344, *p* = 0.006; 40% threshold) times more likely to incur CHE (Tables 7 and 8).

**Table 7:** Logit model coefficients for CHE (20% threshold)—household-level data

Variable	B	Wald	*p*-value	Odds ratio	95% CI for odds ratio
Household with at least one chronically ill member	1.314	18.703	< 0.0001	3.720	2.051–6.704
Household with at least one member hospitalized for more than one night	0.93	9.215	0.002	2.355	1.390–4.620
Household with at least one member who visited a private facility for treatment	0.903	7.831	0.005	2.466	1.310–4.640
Richest: Above 3,500,000 LAK (217.7 USD)/month (reference group)					
Rich: 2,000,000 LAK (124.4 USD)–3,500,000 LAK (217.7 USD)/month	0.148	0.105	0.746	1.160	0.473–2.845
Poor: 1,000,000 LAK (62.2 USD)–2,000,000 LAK (124.4 USD) /month	0.946	5.476	0.019	2.576	1.165–5.688
Poorest: Under 1,000,000 LAK (62.2 USD)/month	1.387	11.923	0.001	4.003	1.821–8.792
Household size < 5	0.755	6.338	0.012	2.127	1.182–3.829
Log likelihood	304.877				
Pseudo R2	0.166				
Hosmer-Lemeshow test	χ2(8) = 5.085				
	*p* = 0.748				
Observations	312

**Table 8:** Logit model coefficients for CHE (40% threshold)—household-level data

Variable	B	Wald	*p*-value	Odds ratio	95% CI for odds ratio
Household with at least one chronically ill member	1.471	16.442	< 0.0001	4.354	2.138–8.866
Household with at least one member hospitalized for more than one night	1.214	12.155	< 0.0001	3.367	1.702–6.663
Richest: Above 3,500,000 LAK (217.7 USD)/month (reference group)					
Rich: 2,000,000 LAK (124.4 USD)–3,500,000 LAK (217.7 USD)/month	0.066	0.013	0.908	1.069	0.345–3.310
Poor: 1,000,000 LAK (62.2 USD)–2,000,000 LAK (124.4 USD)/month	0.662	1.681	0.195	1.945	0.713–5.280
Poorest: Under 1,000,000 LAK (62.2 USD)/month	1.212	5.994	0.014	3.369	1.273–8.872
Household size < 5	0.989	7.471	0.006	2.669	1.321–5.344
Distance to provincial level hospital > 10 km	1.566	8.689	0.003	4.587	1.690–13.558
Log likelihood	234.767				
Pseudo R2	0.147				
Hosmer-Lemeshow test	χ2(8) = 10.074				
	*p* = 0.260				
Observations	314				

## Discussion

Our study clarified the health-seeking behaviors (especially vis-à-vis chronic diseases) and related financial burdens of households in Bolikhamxay province, one of the provinces in the Lao PDR fully covered by the current NHI system. Regarding the positive aspects of the NHI, most households with non-chronically ill (69.9%) and chronically ill (78.8%) patients reported that they visited a health facility for treatment. This study did not clarify whether this trend is a direct effect of the NHI because we did not analyze data from the period preceding the NHI’s implementation. Nevertheless, our findings highlight that in Bolikhamxay province, where the NHI has been implemented, more than 75% of the households in lower-income groups visited a health facility when one of the members had an illness; however, there were some gaps between income groups. Our findings are consistent with two previous studies conducted in a different province [19, 20], which found that the NHI has had positive effects on healthcare accessibility.

Furthermore, this study examined the key financial burdens for households under the current NHI scheme. We observed that 25.9 and 16.2% of the households with at least one member with any self-reported health problem incurred CHE (20%) and serious CHE (40%) per month, respectively. Households with at least one member who was chronically ill or needed hospitalization were more likely to incur CHE. OOP health expenditure for chronic and severe diseases was a considerable burden on households, especially poorer ones; thus, the design of future CHE prevention interventions must take these factors into account [42–45]. Our findings indicate that the financial burden of households under the NHI remains high, especially for those with members who have chronic and/or severe diseases. Moreover, our analysis of household demographics showed that even among households covered by the NHI, OOP health expenditure is greatly polarized between small payments (i.e., within the value covered by the NHI) and very large payments (i.e., which greatly exceed the value covered by the NHI).

We offer the following reasons for these results. First, there are many households that prefer or may be forced to visit private facilities, as our findings showed that households with members who used private facilities were more likely to incur CHE, compared with those who used only public facilities. Our findings are consistent with those of previous studies which showed that people in the Lao PDR preferred to utilize private health services as their first choice regardless of their socioeconomic status, whereas the utilization of public services was low; additionally, high-income households preferred private clinics and receiving treatment abroad [46–48]. Another study found that many people from the Lao PDR sometimes used the health services from certain provinces in Thailand, where most health workers speak Isan Thai (a dialect of the Lao language) as their native tongue [48].

We hypothesize that this may have been because the members had diseases that were difficult to treat or could only be treated in private facilities; alternatively, people may still prefer private facilities even if they are costlier and not covered by the NHI. Therefore, to avoid CHE in Lao households covered by the NHI, managers must ensure that effective treatment for chronic and complex diseases can be provided at facilities covered by the NHI. To achieve this, it is necessary to increase the population’s willingness to visit public facilities, mainly by improving service quality and insurance coverage in public hospitals.

Our results also showed that many households incurred CHE even in public hospitals. This may be due to additional, indirect costs related to visiting health facilities, such as transportation costs, which are not covered by the NHI co-payment scheme. Previous research has also pointed out the existence of additional expenditures for patients visiting public health facilities, including those related to medications or supplies not available in public health facilities or not covered by the NHI [48]. Thus, to prevent CHE among households covered by the NHI, it is necessary to expand the scope of NHI coverage, especially for chronically ill patients (mainly those with NCDs), who require more complex and often prolonged treatments.

Among chronically ill patients in our sample, most visited the provincial, top referral hospital; however, there is only one such hospital in Bolikhamxay province. This denotes that travelling to the hospital entails a high cost (both time and money) for some households [49, 50]. Coupled with the abovementioned CHE rate, this becomes a critical concern for managers. To implement a UHC system, primary care must be emphasized [51]. However, this emphasis on primary care may lead some patients to suffer from complex and/or life-threatening problems if they are not referred to higher level facilities; this is especially true of aging societies such as that of the Lao PDR [52, 53]. These patients require advanced treatment, which often entails high costs that may lead to CHE. Hence, managers should place additional focus on methods for providing NHI patients with advanced care while considering the reality of their financial situation and the constraints of the NHI.

The results of this study, which underscore the differences in medical expenditure across various groups, also highlight the importance of supporting high medical expenditures. For example, in the Japanese NHI, there is a fixed percentage (10–30%) for co-payments for treatment as well as a limit for total co-payments per month, called the “ceiling amount” [54]. We advocate for a similar financial system for the NHI in the Lao PDR, which may help prevent CHE in households with chronically ill members.

To implement UHC, it is important to enhance the coverage of healthcare systems, as well as citizens’ financial protection. In the Lao PDR, the NHI was initially introduced with the aim of covering the entire population of select provinces, as well as to provide financial protection through a fixed payment system. However, it did not necessarily consider the enhancement of service provision; thus, we believe that the NHI’s potential preventive effect on CHE has been limited.

Even in poor households, it is common for people to seek better medical services when they become ill. Our findings underpin that CHE is more likely to occur in households with lower incomes. Similarly, global studies have highlighted the importance of ensuring a reasonable distribution of health services across different community-based and socioeconomic strata, especially for patients with chronic diseases [49]. Accordingly, our discussion suggests that the NHI requires additional improvements regarding health facility placement; patients must be able to access reasonably located healthcare facilities that deliver quality services. This should also be valid for facilities aimed at treating chronically ill patients, which often require long-term and frequent care delivery. To realize UHC in the Lao PDR, the government must adopt a comprehensive approach to the NHI that better reflects the current situation.

This study has some limitations. First, we analyzed data on households’ finances (i.e., health expenditure and income) by interviewing the household heads; however, this method may have affected data precision. Second, self-reported diagnoses were included for citizens who did not visit a health facility; however, the estimates (prevalence) were not weighted to obtain province-representative evidence and a longitudinal design was not adopted to assess the difference between the pre- and post-NHI periods.

Nevertheless, to the best of our knowledge, this is the first study to investigate the health-seeking behaviors and financial burdens of households covered by the NHI in the Lao PDR, including those with chronically ill patients. Most previous studies on the NHI in the Lao PDR focused on patients who visited healthcare facilities; consequently, their data did not consider patients who did not visit or could not access such facilities. Therefore, we deemed it necessary to examine the situation of people who did not or could not visit healthcare facilities; this allowed us to assess the effectiveness of interventions aimed at realizing UHC, such as the NHI in the Lao PDR. This is because the main purpose of the NHI is to improve the general population’s access to health facilities while ensuring they do not incur major financial burdens related to healthcare.

## Conclusion

Our results provide a clear picture of the current status of the general population in provinces covered by the NHI, particularly regarding access to health services and households’ financial burden, especially for those with chronically ill members. We hope that this evidence provides a valuable theoretical framework for managers and policymakers to conduct well-informed decision-making to facilitate the realization of UHC in the Lao PDR.

## Data Availability

The data supporting the conclusions of this article are available from the corresponding author upon reasonable request.

## Abbreviations

NCDs: Non-communicable diseases
NHI: National Health Insurance
UHC: Universal health coverage
CHE: Catastrophic health expenditure
OOP: Out-of-pocket
