# Correction: Financial burden and health-seeking behaviors related to chronic diseases under the National Health Insurance Scheme in Bolikhamxay Province, Lao PDR: a cross-sectional study

**DOI:** 10.1186/s12939-023-01827-4

**Published:** 2023-02-23

**Authors:** Tomoo Ito, Sengchanh Kounnavong, Chiaki Miyoshi

**Affiliations:** 1 Bureau of International Health Cooperation, National Center for Global Health and Medicine, 1-21-1 Toyama Shinjuku-ku, Tokyo, 162-8655 Japan; 2 Lao Tropical and Public Health Institute, Ministry of Health, Samsenethai Road, Ban Kaognot, Sisattanack District, Vientiane Capital Lao PDR

**Correction: Int J Equity Health 21, 180 (2022)**

**https://doi.org/10.1186/s12939-022-01788-0**

After publication of this article [1], the authors reported that in Table 7 the odds ratio for ‘Household size < 5’ was incorrectly given as '2.127' but should have been '2.218', and in Table 8 the odds ratio for ‘Poorest: Under 1,000,000 LAK (62.2 USD)/month’ was incorrectly given as '3.369' but should have been '3.339'.

The original article [1] has been corrected.

## References

[1] Ito T (2022). Financial burden and health-seeking behaviors related to chronic diseases under the National Health Insurance Scheme in Bolikhamxay Province, Lao PDR: a cross-sectional study. Int J Equity Health..

